# Comparative Evaluation of Automated Retreatment Systems for Bioceramic Sealer Removal: An In Vitro Scanning Electron Microscopic Study

**DOI:** 10.7759/cureus.112977

**Published:** 2026-07-19

**Authors:** Sufia Parveen, Shalini Kumari, Chi Koy Wang, Abhinav Kumar Singh, Somnath Pal, Binoy Kumar Singh, Navin Mishra

**Affiliations:** 1 Department of Conservative Dentistry and Endodontics, Buddha Institute of Dental Sciences and Hospital, Patna, IND; 2 Department of Conservative Dentistry and Endodontics, Post Graduate Institute of Dental Education and Research (PGIDER), Indira Gandhi Institute of Medical Sciences, Patna, IND

**Keywords:** bioceramic sealer, endodontic retreatment, hyflex remover, neoendo retreatment, protaper universal retreatment, root canal sealer, scanning electron microscopy

## Abstract

Background: Calcium silicate-based bioceramic sealers have gained widespread acceptance because of their excellent sealing ability and biocompatibility. However, their strong adhesion to dentinal walls poses a significant challenge during nonsurgical endodontic retreatment. This study evaluated and compared the efficacy of the Neoendo Retreatment (Neoendo, Orikam Healthcare India Pvt. Ltd., Gurugram, India), ProTaper Universal Retreatment (Dentsply Sirona, Ballaigues, Switzerland), and HyFlex Remover (COLTENE/Whaledent AG, Altstätten, Switzerland) file systems in removing bioceramic sealers from root canal walls using scanning electron microscopy (SEM).

Materials and methods: This in vitro study evaluated 45 extracted human mandibular premolars obturated with a calcium silicate-based bioceramic sealer and thermoplasticized gutta-percha. After standardized root canal preparation and obturation, the specimens were randomly allocated into three groups (n = 15) according to the retreatment system used: Neoendo Retreatment, ProTaper Universal Retreatment, and HyFlex Remover. Following retreatment, the roots were longitudinally sectioned and examined under scanning electron microscopy (SEM) at ×1000 magnification. The presence of residual bioceramic sealer in the coronal, middle, and apical thirds was assessed using the modified Ezzie scoring system. Data were analyzed using the Friedman and Kruskal-Wallis tests, followed by Dunn's multiple comparisons test, where appropriate, with statistical significance set at p < 0.05.

Results: All three retreatment systems left residual bioceramic sealer on the canal walls, and none achieved complete removal. Within each retreatment system, residual bioceramic sealer scores differed significantly among the coronal, middle, and apical thirds (Friedman test, p < 0.05). Dunn's post hoc analysis demonstrated significantly higher residual sealer scores in the apical third than in the coronal third, whereas no significant differences were observed between the coronal and middle thirds or between the middle and apical thirds. Intergroup comparisons revealed no statistically significant differences among the Neoendo Retreatment, ProTaper Universal Retreatment, and HyFlex Remover systems at any root canal level (p > 0.05), although the HyFlex Remover system showed a trend toward lower residual sealer scores. SEM analysis demonstrated region-specific differences in canal cleanliness, characterized by cleaner dentinal surfaces in the coronal third and greater residual bioceramic sealer with marked dentinal tubule occlusion in the apical third.

Conclusion: Neoendo Retreatment, ProTaper Universal Retreatment, and HyFlex Remover exhibited comparable efficacy in bioceramic sealer removal. Complete sealer removal remains unattainable, particularly in the apical third, highlighting the need for adjunctive cleaning strategies to improve the retreatment outcomes.

## Introduction

Successful endodontic treatment relies on effective chemomechanical debridement, elimination of microorganisms, and three-dimensional obturation to prevent reinfection. Despite advances in instrumentation, irrigation, and obturation materials, treatment failure may still occur because of persistent intraradicular infection, untreated canal complexities, procedural errors, or inadequate sealing of the root canal system. In such cases, nonsurgical endodontic retreatment remains the preferred treatment option, aiming to eliminate the cause of failure while preserving natural dentition and promoting periapical healing [1]. A critical objective of retreatment is the complete removal of existing root canal filling materials to facilitate reinstrumentation, disinfection, and re-obturation procedures. However, residual gutta-percha and sealer frequently persist within canal irregularities and dentinal tubules, compromising retreatment outcomes [2].

Calcium silicate-based bioceramic sealers have gained widespread clinical acceptance because of their excellent physicochemical and biological properties, including hydrophilicity, dimensional stability, biocompatibility, and bioactivity. Their ability to chemically bond with radicular dentin through hydroxyapatite formation enhances the long-term sealing of the root canal system and promotes periapical healing [3,4]. However, these same properties make retreatment considerably more difficult because of their deep penetration into the dentinal tubules, biomineralization, and limited solubility in conventional endodontic solvents [5]. Mechanical instrumentation is the cornerstone of nonsurgical retreatment. Although contemporary nickel-titanium (NiTi) rotary and reciprocating systems have improved retreatment efficiency, complete removal of filling materials remains difficult, particularly in the apical third and anatomically complex regions [6-9]. Likewise, sodium hypochlorite and ethylenediaminetetraacetic acid (EDTA) improve canal cleanliness but cannot dissolve set bioceramic sealers, making mechanical instrumentation indispensable [10,11]. Among the available evaluation methods, scanning electron microscopy (SEM) provides high-resolution assessment of residual sealer and canal cleanliness, making it a reliable tool for evaluating retreatment efficacy [12]. Although several studies have investigated the retreatability of calcium silicate-based sealers, the available evidence remains inconclusive because of variations in instrumentation systems, retreatment protocols, irrigation regimens, and evaluation methods. Furthermore, direct comparisons of contemporary automated retreatment systems using standardized experimental conditions and SEM analysis are limited. Therefore, the present in vitro study was undertaken to compare the efficacy of the Neoendo Retreatment (Neoendo, Orikam Healthcare India Pvt. Ltd., Gurugram, India), ProTaper Universal Retreatment (Dentsply Sirona, Ballaigues, Switzerland), and HyFlex Remover (COLTENE/Whaledent AG, Altstätten, Switzerland) systems in removing bioceramic sealer from root canal walls using SEM through the evaluation of residual bioceramic sealer.

## Materials and methods

Study design

This in vitro experimental study was conducted to evaluate and compare the efficacy of different automated retreatment instrumentation systems in removing calcium silicate-based bioceramic sealer from root canal walls using scanning electron microscopy (SEM). This study was conducted at the Department of Conservative Dentistry and Endodontics, Buddha Institute of Dental Sciences and Hospital, Patna, Bihar, in collaboration with the Department of Mechanical Engineering, Indian Institute of Technology (IIT), Patna, Bihar, where the scanning electron microscopic (SEM) analysis was performed. The study protocol was approved by the Institutional Ethics Committee (IEC-BIDSH) of the Buddha Institute of Dental Sciences and Hospital, Patna, Bihar, India (approval number: 80/BIDSH/IEC/EXEMP/2024).

Sample size calculation

The sample size was calculated using G*Power software version 3.1 (Heinrich Heine University, Düsseldorf, Germany). Based on an effect size (f) of 0.508, alpha error probability of 0.05, study power of 80%, and three study groups, the minimum required sample size was calculated as 42 specimens [13]. To compensate for possible specimen loss during specimen preparation and SEM analysis, the sample size was increased to 45 teeth, with 15 specimens in each study group.

Sample selection

A total of 45 freshly extracted human permanent mandibular premolars extracted for orthodontic purposes were collected from the Department of Oral and Maxillofacial Surgery of our institution. All teeth were examined clinically and radiographically to confirm the presence of a single, straight root canal with a completely formed apex.

Inclusion criteria

Extracted human permanent mandibular premolars with a single straight root canal and fully developed apices were included in the study. Only teeth extracted for orthodontic purposes with intact roots and no history of endodontic treatment were selected. Clinical and radiographic examinations were performed to confirm the presence of a single canal with less than 20-degree curvature and complete root formation before inclusion.

Exclusion criteria

Teeth with previous endodontic treatment, root caries, fractures, cracks, root resorption, developmental anomalies, open apices, severe root curvature, calcified canals, or any structural defects that could influence instrumentation or retreatment procedures were excluded from the study.

Following extraction, soft tissue remnants and calculus were removed using ultrasonic scalers (Woodpecker Medical Instrument Co., Ltd., Guilin, China). The teeth were cleaned and stored in normal saline at room temperature until use to prevent their dehydration.

Root canal preparation

The coronal portions of the teeth were sectioned using a double-sided diamond disc (MANI Inc., Tochigi, Japan) under continuous water irrigation to obtain standardized root specimens measuring 16 mm in length. Canal patency was established using a #10 K-file (MANI Inc., Tochigi, Japan), and the working length was determined using radiovisiography (RVG) by subtracting 1 mm from the radiographic length. Biomechanical preparation was performed using the ProTaper Gold nickel-titanium rotary file system (Dentsply Sirona, Ballaigues, Switzerland) up to size F2 in a crown-down sequence using an E-Connect endodontic motor (Eighteeth Medical Technology Co., Ltd., Changzhou, China). During instrumentation, each canal was irrigated with 2 mL of 5.25% sodium hypochlorite (HypoChlor; Septodont Healthcare India Pvt. Ltd., India) after every instrument change. Following instrumentation, the canals were irrigated with 5 mL of 17% EDTA solution (Waldent Innovations Pvt. Ltd., New Delhi, India), followed by 5 mL of normal saline, and dried with sterile absorbent paper points before obturation.

Root canal obturation

The prepared canals were obturated using thermoplasticized injectable gutta-percha with the Fast-Pack FI-G obturation system (Eighteeth Medical Technology Co., Ltd., Changzhou, China) in conjunction with a calcium silicate-based bioceramic root canal sealer (Bio-C Sealer; Angelus, Londrina, Paraná, Brazil), according to the manufacturer's instructions. The quality of the obturation was verified radiographically to ensure complete filling without voids. All specimens were stored at 37°C and 100% relative humidity until the bioceramic sealer was completely set before retreatment procedures were initiated.

Group allocation

Following the complete setting of the sealer, the specimens were randomly allocated using a computer-generated randomization sequence into three equal groups (n = 15) according to the retreatment instrument system used.

Group I: Neoendo Retreatment File System

Retreatment was performed using the Neoendo rotary retreatment system (Neoendo, Orikam Healthcare India Pvt. Ltd., Gurugram, India), which consists of sequential N1, N2, and N3 files. The instruments were used in a crown-down sequence from the coronal to the apical third until the working length was reached, and no visible filling material remained on the instruments.

Group II: ProTaper Universal Retreatment File System

Retreatment was performed using the ProTaper Universal Retreatment System (Dentsply Maillefer, Ballaigues, Switzerland), which comprises D1, D2, and D3 files. The D1 file was used for the coronal third, D2 for the middle third, and D3 for the apical third, respectively, following the manufacturer's recommended protocol until complete removal of the filling material was attempted.

Group III: HyFlex Remover File System

Retreatment was performed using a HyFlex Remover file (COLTENE/Whaledent AG, Altstätten, Switzerland) operated in continuous rotary motion according to the manufacturer's instructions. The instrument was gradually advanced to the working length until no visible obturation material was observed on the instrument flutes upon withdrawal.

During retreatment, all specimens, irrespective of the study group, were irrigated using an identical protocol consisting of intermittent irrigation with 2 mL 5.25% sodium hypochlorite after each instrument sequence. The same irrigation regimen was maintained for Groups I, II, and III to ensure the standardization of the retreatment procedure. Retreatment was considered complete when the working length was reached and no visible gutta-percha remnants were present on the instruments.

Preparation of specimens for scanning electron microscopy

After retreatment, all specimens were longitudinally sectioned using a water-cooled diamond disc. Each root was carefully split into two halves without disturbing the residual filling material that adhered to the canal walls. The specimens were dehydrated in ascending concentrations of ethanol (50%, 70%, 80%, 90%, and 100%) for approximately 10-15 minutes at each concentration. After dehydration, the specimens were air-dried and mounted on aluminum stubs. A thin layer of gold was deposited over the specimens using a sputter coater to render the surface electrically conductive prior to the SEM examination.

Scanning electron microscopy evaluation

Scanning electron microscopy (SEM) evaluation was performed using a Gemini SEM 500 (ZEISS Microscopy GmbH, Oberkochen, Germany) at the Department of Mechanical Engineering, Indian Institute of Technology (IIT), Patna. Before examination, the specimens were gold sputter-coated using a Q150 Series sputter coater (Quorum Technologies Ltd., Laughton, East Sussex, United Kingdom) and examined at ×1000 magnification. SEM images were obtained from the coronal (9 mm from the apex), middle (6 mm from the apex), and apical thirds (3 mm from the apex) of each root canal. To minimize operator-related variability, a single experienced operator performed all retreatment procedures, and a single blinded examiner evaluated the SEM images under standardized conditions. Residual bioceramic sealer was assessed using the modified scoring system of Ezzie et al. [14]: score 1 (0%-25%), score 2 (25%-50%), score 3 (50%-75%), and score 4 (>75%) residual sealer. The coronal, middle, and apical thirds were evaluated separately, and the scores were analyzed using appropriate non-parametric statistical tests.

Statistical analysis

The collected data were entered into Microsoft Excel (Microsoft Corporation, Redmond, WA) and analyzed using GraphPad Prism version 8.4.3 (GraphPad Software, Boston, MA). As the residual bioceramic sealer scores were based on an ordinal four-point scoring system, non-parametric statistical tests were used. Data are expressed as median and interquartile range (IQR). Intragroup comparisons of residual bioceramic sealer scores among the coronal, middle, and apical thirds were performed using the Friedman test, followed by Dunn's multiple comparisons test to identify pairwise differences. Intergroup comparisons among the three retreatment systems at each root canal level were performed using the Kruskal-Wallis test. A p-value < 0.05 was considered statistically significant.

## Results

A total of 45 extracted human mandibular premolars were included in this study, with 15 specimens allocated to each retreatment group. All specimens completed the retreatment and SEM evaluation protocols, and none were excluded from the final analysis. The residual bioceramic sealer was assessed separately in the coronal (9 mm from the apex), middle (6 mm from the apex), and apical thirds (3 mm from the apex) of each root canal, using the modified Ezzie scoring system.

The median residual bioceramic sealer scores at the coronal, middle, and apical thirds for each retreatment system are presented in Table 1. Intragroup analysis using the Friedman test demonstrated statistically significant differences among the three root canal levels within the Neoendo Retreatment (χ² = 9.50, p = 0.0087), ProTaper Universal Retreatment (χ² = 8.71, p = 0.0128), and HyFlex Remover (χ² = 9.81, p = 0.0074) groups (Table 1), demonstrating statistically significant differences in residual bioceramic sealer scores among the coronal, middle, and apical thirds.

**Table 1:** Intragroup Comparison of Residual Bioceramic Sealer Scores at the Coronal, Middle, and Apical Thirds Within Each Retreatment File System Data are presented as median (IQR). Intragroup comparisons were performed using the Friedman test. Statistical significance was set at p < 0.05. χ² = Friedman test statistic *Statistically significant IQR: interquartile range

Group	Coronal median (IQR)	Middle median (IQR)	Apical median (IQR)	Friedman χ²	p-value
Group I (Neoendo Retreatment)	2 (1-2)	2 (2-3)	3 (2-3)	9.50	0.0087*
Group II (ProTaper Universal Retreatment)	2 (1-2)	2 (2-2)	2 (2-3)	8.71	0.0128*
Group III (HyFlex Remover)	1 (1-2)	2 (1-2)	2 (2-3)	9.81	0.0074*

Pairwise intragroup comparisons using Dunn's multiple comparisons test (Table 2) demonstrated statistically significant differences between the coronal and apical thirds in the Neoendo Retreatment (adjusted p = 0.0140), ProTaper Universal Retreatment (adjusted p = 0.0318), and HyFlex Remover (adjusted p = 0.0243) groups. No statistically significant differences were observed between the coronal and middle thirds or between the middle and apical thirds for any of the retreatment systems (all adjusted p > 0.05).

**Table 2:** Pairwise Intragroup Comparison of Residual Bioceramic Sealer Scores Among the Coronal, Middle, and Apical Thirds Within Each Retreatment File System Data are presented as adjusted p-values obtained using Dunn's multiple comparisons test following the Friedman test. Statistical significance was set at p < 0.05. *Statistically significant

Group	Comparison	Adjusted p-value
Group I (Neoendo Retreatment)	Coronal versus middle	0.9459
	Coronal versus apical	0.0140*
	Middle versus apical	0.2037
Group II (ProTaper Universal Retreatment)	Coronal versus middle	0.6037
	Coronal versus apical	0.0318*
	Middle versus apical	0.6037
Group III (HyFlex Remover)	Coronal versus middle	0.4324
	Coronal versus apical	0.0243*
	Middle versus apical	0.7060

Intergroup comparison using the Kruskal-Wallis test (Table 3) demonstrated no statistically significant differences among the Neoendo Retreatment, ProTaper Universal Retreatment, and HyFlex Remover systems in the coronal (H = 4.14, p = 0.126), middle (H = 2.64, p = 0.267), or apical (H = 3.88, p = 0.144) thirds, indicating that all three retreatment systems exhibited comparable efficacy in removing bioceramic sealer from root canal walls.

**Table 3:** Intergroup Comparison of Residual Bioceramic Sealer Scores at Different Root Canal Levels Using the Kruskal-Wallis Test Intergroup comparisons among the three retreatment file systems were performed using the Kruskal-Wallis test. Statistical significance was set at p < 0.05. NS: not statistically significant

Level	Kruskal-Wallis H	p-value	Significance
Coronal (9 mm)	4.14	0.126	NS
Middle (6 mm)	2.64	0.267	NS
Apical (3 mm)	3.88	0.144	NS

SEM observations

**Figure 1:**
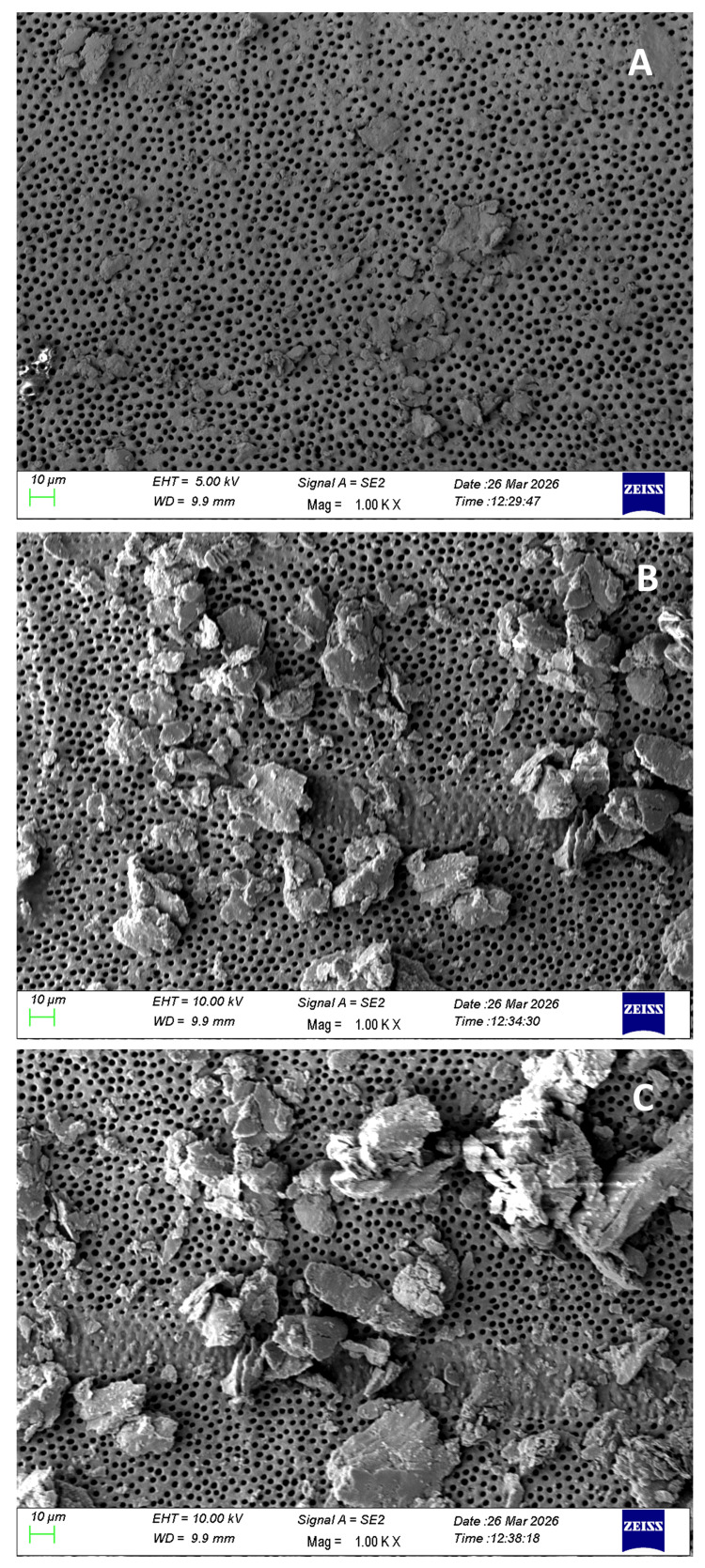
Representative SEM Images of Group I (Neoendo Retreatment File System) at ×1000 Magnification: A, Coronal Third; B, Middle Third; and C, Apical Third SEM: scanning electron microscopy

**Figure 2:**
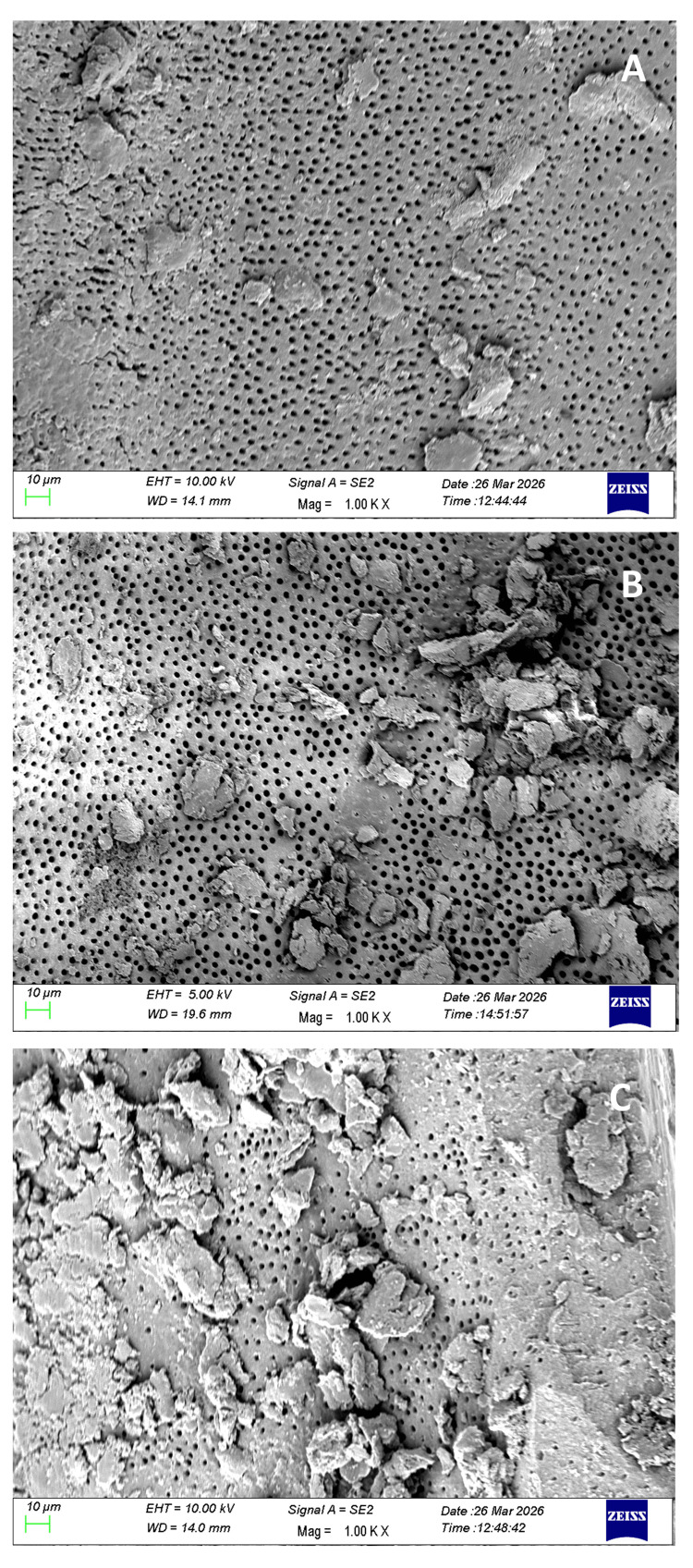
Representative SEM Images of Group II (ProTaper Universal Retreatment System) at ×1000 Magnification: A, Coronal Third; B, Middle Third; and C, Apical Third SEM: scanning electron microscopy

**Figure 3:**
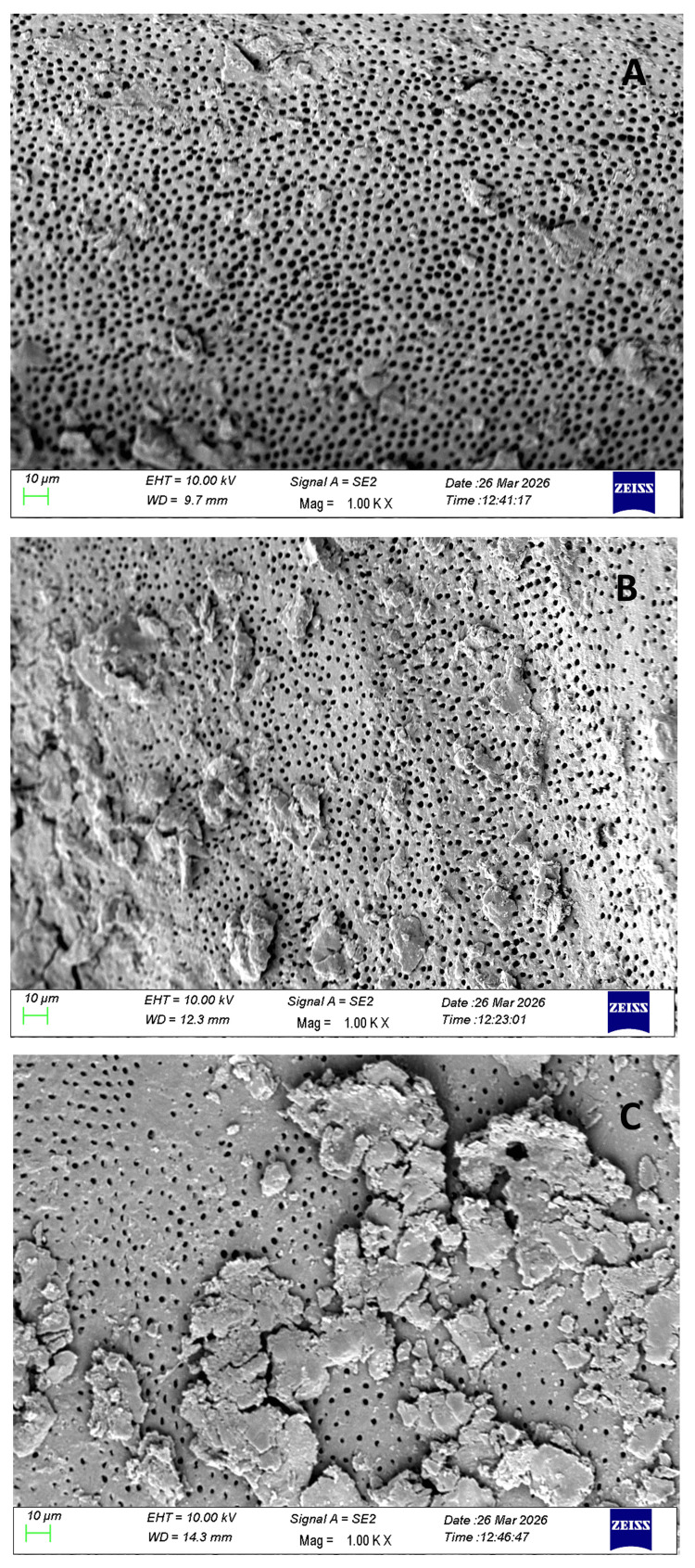
Representative SEM Images of Group III (HyFlex Remover File System) at ×1000 Magnification: A, Coronal Third; B, Middle Third; and C, Apical Third SEM: scanning electron microscopy

Scanning electron microscopy evaluation revealed distinct variations in the amount and distribution of residual bioceramic sealer at different levels of the root canal in all three retreatment systems. Representative SEM images of the Neoendo Retreatment, ProTaper Universal Retreatment, and HyFlex Remover systems are presented in Figures 1-3, respectively. In the Neoendo Retreatment group, the coronal third exhibited relatively clean canal walls with minimal residual sealer and widely patent dentinal tubules (Figure 1A), whereas the middle third demonstrated moderate residual sealer with partial occlusion of the dentinal tubules (Figure 1B). The apical third showed the greatest accumulation of residual bioceramic sealer and debris with marked obliteration of the dentinal tubules (Figure 1C). Similar observations were made in the ProTaper Universal Retreatment group, with cleaner canal walls in the coronal third (Figure 2A), moderate residual sealer in the middle third (Figure 2B), and the highest amount of residual sealer and debris in the apical third (Figure 2C). In the HyFlex Remover group, comparatively cleaner canal walls with less residual sealer were observed in the coronal (Figure 3A) and middle thirds (Figure 3B), while residual sealer and debris remained evident in the apical third with marked dentinal tubule obliteration (Figure 3C). Overall, SEM examination demonstrated cleaner dentinal surfaces in the coronal third and greater residual bioceramic sealer with marked dentinal tubule occlusion in the apical third. Although the HyFlex Remover system demonstrated comparatively cleaner canal walls with less residual sealer than the ProTaper Universal Retreatment and Neoendo Retreatment systems, these differences were not statistically significant. None of the tested retreatment systems achieved complete removal of the bioceramic sealer at any level of the root canal.

## Discussion

The present in vitro study evaluated and compared the efficacy of three automated retreatment systems, Neoendo Retreatment File System (Group I), ProTaper Universal Retreatment System (Group II), and HyFlex Remover File System (Group III), for the removal of bioceramic sealers from root canal walls using scanning electron microscopy (SEM). Although all three systems effectively removed a substantial amount of filling material, none achieved complete elimination of the bioceramic sealers. These findings suggest a persistent challenge associated with the retreatment of calcium silicate-based sealers because of their chemical bonding with dentin and biomineralization properties.

The intragroup analysis demonstrated significantly higher residual bioceramic sealer scores in the apical third than in the coronal third for all three retreatment systems. This observation is consistent with previous studies reporting that complete removal of obturation materials is difficult, particularly in the apical region, because of anatomical complexities and limited instrument and irrigant penetration [15]. Similarly, Al Akam et al. (2024) [16] concluded that although rotary and reciprocating NiTi systems improve retreatment efficiency, no currently available technique can completely remove the bioceramic sealers.

The present study found no statistically significant differences in residual bioceramic sealer scores among the Neoendo Retreatment, ProTaper Universal Retreatment, and HyFlex Remover systems. These findings suggest that all three systems exhibit comparable efficacy for bioceramic sealer removal under the experimental conditions of this study. Comparable findings were reported by Abdelnaby et al. (2023) [15], who observed no significant differences among contemporary retreatment systems. In contrast, Mann et al. (2023) [17] reported a superior performance of ProTaper Universal Retreatment over Neoendo Retreatment, whereas Karunakar et al. (2024) [18] found that HyFlex left the least residual filling material. These variations may be attributed to differences in canal anatomy, sealer types, evaluation methods, and retreatment protocols.

Intragroup analysis using the Friedman test, followed by Dunn's multiple comparisons test, demonstrated significantly higher residual bioceramic sealer scores in the apical third than in the coronal third for all three retreatment systems. However, no significant differences were observed between the coronal and middle thirds or between the middle and apical thirds of the canal. The reduced cleaning efficiency in the apical region may be explained by its smaller diameter, anatomical irregularities, restricted irrigant exchange, and deeper penetration of bioceramic sealers into the dentinal tubules. Similar observations have been reported by Bramhecha et al. (2023) [12] and Kakoura and Pantelidou (2018) [2], who also found greater residual filling material in the apical third after retreatment.

SEM examination revealed cleaner dentinal surfaces with open dentinal tubules in the coronal third, partial tubule occlusion in the middle third, and greater residual bioceramic sealer with marked dentinal tubule occlusion in the apical third. These observations confirm that mechanical instrumentation alone is insufficient to completely remove bioceramic sealers, regardless of the file system employed.

The difficulty in removing bioceramic sealers is related to their hydrophilic nature, hydroxyapatite formation, chemical adhesion to dentin, and limited solubility in conventional endodontic solvents. Previous investigations have similarly reported that calcium silicate-based sealers are more difficult to retrieve than conventional epoxy resin-based sealers [19,20], although some studies have shown variable retreatability among bioceramic sealers [5,21].

From a clinical perspective, the findings suggest that the retreatment of teeth obturated with bioceramic sealers should not rely solely on mechanical instrumentation. Adjunctive procedures, such as supplementary finishing files, ultrasonic activation, laser-activated irrigation, or advanced irrigation protocols, may further enhance the removal of residual filling material, as demonstrated by Farrayeh et al. (2023) [13], Yang et al. (2021) [22], Pedullà et al. (2019) [23], Kasam and Mariswamy (2016) [24], and Lin et al. (2026) [25].

Overall, the present study demonstrated that the Neoendo Retreatment, ProTaper Universal Retreatment, and HyFlex Remover systems showed comparable efficacy in removing the bioceramic sealer. None of the systems completely eliminated the sealer, and the residual material was consistently greater in the apical third. These findings emphasize the need for adjunctive cleaning techniques to improve the predictability of nonsurgical endodontic retreatment involving bioceramic sealers.

Limitations of the study

Although the present in vitro study was conducted under standardized experimental conditions, certain limitations should be considered when interpreting the findings. The use of single-rooted teeth with straight canals may not fully replicate the anatomical complexities encountered in clinical practice. Scanning electron microscopy (SEM) provides high-resolution surface evaluation of residual bioceramic sealers; however, the ordinal scoring system offers a semi-quantitative assessment and does not permit three-dimensional volumetric analysis, as can be achieved with micro-computed tomography (micro-CT). In addition, clinically relevant parameters such as retreatment time, apical debris extrusion [15,26], and the influence of advanced irrigant activation techniques were not investigated in this study. Future studies incorporating more complex root canal anatomies, different bioceramic sealers, volumetric imaging techniques, and clinical outcome measures are warranted to validate and expand upon the present findings.

## Conclusions

The Neoendo Retreatment, ProTaper Universal Retreatment, and HyFlex Remover systems demonstrated comparable efficacy in removing bioceramic sealers from root canal walls, with no statistically significant differences among the three systems. None of the systems achieved complete sealer removal, and the apical third remained the most challenging region for retreatment. These findings underscore the limitations of mechanical retreatment alone and support the incorporation of adjunctive cleaning approaches to improve bioceramic sealer removal and optimize the outcome of nonsurgical endodontic retreatment. Future studies should evaluate advanced irrigation activation methods and other supplementary techniques under clinically relevant conditions.
